# Novel loss of function mutation in *NOTCH1* in a family with bicuspid aortic valve, ventricular septal defect, thoracic aortic aneurysm, and aortic valve stenosis

**DOI:** 10.1002/mgg3.1437

**Published:** 2020-07-27

**Authors:** Radoslaw Debiec, Stephen E. Hamby, Peter D. Jones, Sue Coolman, Manish Asiani, Shireen Kharodia, Gregory J. Skinner, Nilesh J. Samani, Tom R. Webb, Aidan Bolger

**Affiliations:** ^1^ Department of Cardiovascular Sciences University of Leicester NIHR (National Institute for Health Research) Leicester Biomedical Research Centre Glenfield Hospital Leicester UK; ^2^ East Midlands Congenital Heart Centre Glenfield Hospital Leicester UK

**Keywords:** bicuspid aortic valve, exome sequencing, NOTCH1

## Abstract

**Background:**

Bicuspid aortic valve is the most common congenital valvular heart defect in the general population. BAV is associated with significant morbidity due to valve failure, formation of thoracic aortic aneurysm, and increased risk of infective endocarditis and aortic dissection. Loss of function mutations in *NOTCH1* (OMIM 190198) has previously been associated with congenital heart disease involving the aortic valve, left ventricle outflow tract, and mitral valve that segregates in affected pedigrees as an autosomal dominant trait with variable expressivity.

**Methods:**

We performed whole‐exome sequencing in four members of a three‐generational family (three affected and one unaffected subject) with clinical phenotypes including aortic valve stenosis, thoracic aortic aneurysm, and ventricular septal defect.

**Results:**

We identified 16 potentially damaging genetic variants (one stop variant, one splice variant, and 14 missense variants) cosegregating with the phenotype. Of these variants, the nonsense mutation (p.Tyr291*) in *NOTCH1* was the most deleterious variant identified and the most likely variant causing the disease.

**Conclusion:**

Inactivating *NOTCH1* mutations are a rare cause of familial heart disease involving predominantly left ventricular outflow tract lesions and characterized by the heterogeneity of clinical phenotype.

## Graphical abstract

Exome sequencing in four members of a three‐generational family (three affected and one unaffected subject) with clinical phenotypes including aortic valve stenosis, thoracic aortic aneurysm, and ventricular septal defect revealed nonsense mutation (p.Tyr291*) in NOTCH1. Inactivating NOTCH1 mutations are a rare cause of familial heart disease involving predominantly left ventricular outflow tract lesions and characterized by heterogeneity of clinical phenotype.

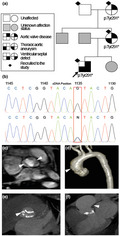

## 1 INTRODUCTION

Bicuspid aortic valve (BAV), which affects 1%–2% of the general population, is a congenital heart defect where the aortic valve consists of two rather than three separate leaflets (Mordi & Tzemos, 2012). BAV is associated with significant morbidity in later life due to aortic valve stenosis, regurgitation or infective endocarditis, and the majority of patients with BAV will require aortic valve replacement (Michelena et al., 2008). BAV normally follows an autosomal dominant inheritance pattern with reduced penetrance. Individuals with BAV are also at greater risk of thoracic aortic aneurysm (TAA) and aortic dissection (Michelena et al., 2011). Mutations in several genes including *NOTCH1 (OMIM 190198)*, *SMAD6 (OMIM 602931)*, and *ROBO4 (OMIM 607528)* have been shown to cause BAV; however, the number of BAV families where the disease‐causing mutation has been identified remains relatively low (Garg et al., 2005; Gillis et al., 2017; Gould et al., 2019).

*NOTCH1* is the gene with the strongest evidence for causative association with congenital aortic valve disease including BAV. *NOTCH1* encodes a transmembrane receptor involved in developmental processes and cell fate decisions. Aside from BAV, mutations in *NOTCH1* have been reported to associate with other congenital cardiac lesions including congenital aortic valve stenosis, coarctation of aorta, and hypoplastic left heart syndrome and, less frequently, right‐sided cardiac lesions (Freylikhman et al., 2014; Helle et al., 2019; Kerstjens‐Frederikse et al., 2016; Southgate et al., 2015). Evidence also exist to link *NOTCH1* genetic variants with tricuspid aortic valve calcification leading to aortic stenosis and development of ascending thoracic aneurysm (Acharya et al., 2011; Ducharme et al., 2013).

Here, we report the whole‐exome sequencing in members of a nuclear pedigree affected by BAV, ventricular septal defect (VSD), aortic valve stenosis, and TAA segregating with a pathogenic *NOTCH1* mutation.

## 2 MATERIALS AND METHODS

### 2.1 Ethical compliance

The study protocol was approved by the local Research Ethics Committee and the study was performed in accordance with the Good Clinical Practice Standards. All subjects participating in the BRAVE study provided informed consent for the study procedures, use of clinical data, and publication of results.

### 2.2 Study subjects

The subjects of the study were members of a nuclear family recruited as part of the University of Leicester Bicuspid aoRtic vAlVe gEnetic research (BRAVE) study; an ongoing collection of patients diagnosed with BAV. For patients who consented for participation, clinical data were obtained from research questionnaires, medical notes, imaging studies, and operation notes. Relatives with unknown affection status underwent transthoracic echocardiogram to assess aortic valve status.

### 2.3 DNA isolation, quality control, and exome sequencing

**Figure 1:**
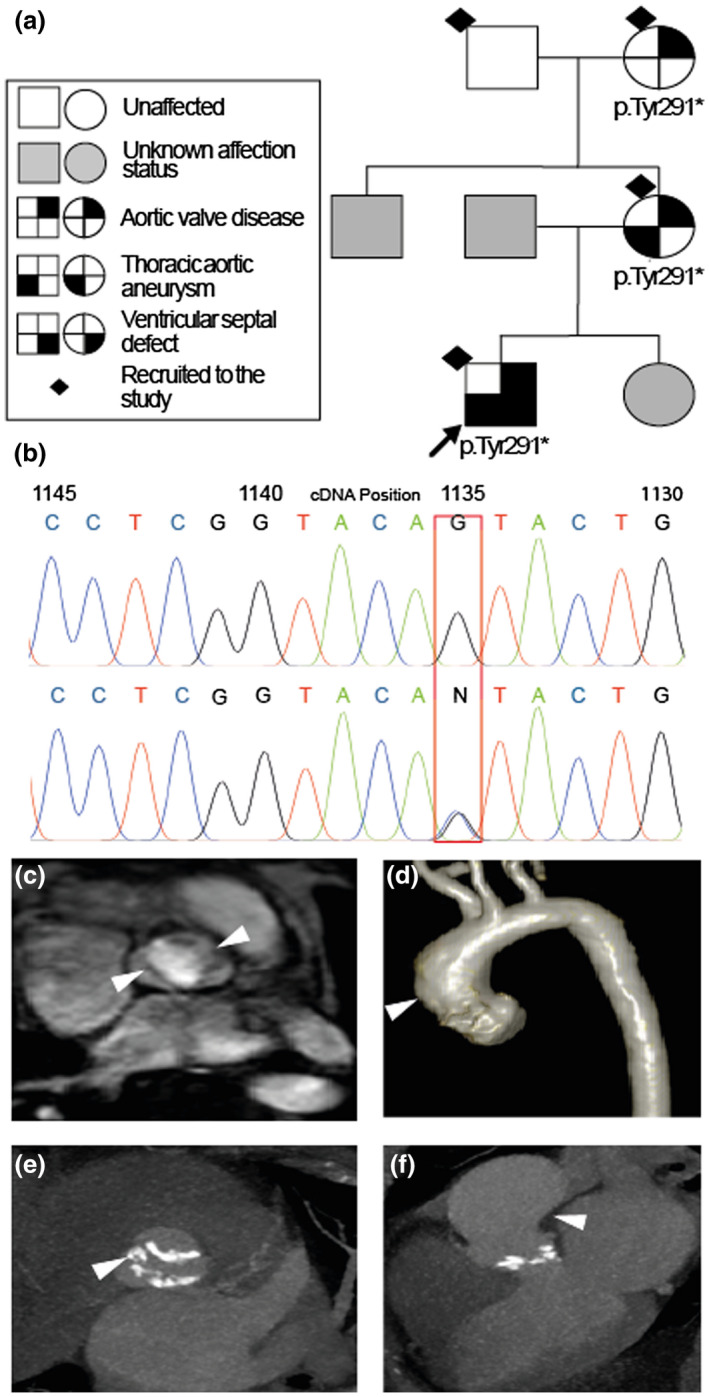
Clinical phenotypes observed in the pedigree. (a) Pedigree of the affected family and phenotype key; squares represent males, circles represent females; proband is indicated with black arrow; phenotypes are marked in solid black in the relevant quadrant of the figure; solid grey represents family members of unknown affection status not recruited to the study; black diamond represents subjects with available DNA; p.Tyr291* identifies subjects positive for the mutation in *NOTCH1* (NM_017617.5). (b) Sanger sequence trace; normal sequence from the maternal grandfather of the proband (top) and the heterozygous c.873C>G variant in the proband (bottom). (c) MRI picture of the bicuspid aortic valve in the proband—two leaflets marked with arrows. (d) Reconstruction of the thoracic aorta in the proband (note dilatation of the ascending aorta—arrow). (e) Image of the calcified aortic valve in the proband's mother; calcium deposits marked with an arrow. (f) Image of the calcified aortic valve and dilatation of the ascending aorta (arrow) in the proband's mother

Samples of blood (EDTA) were obtained from each participant. Automated DNA extraction was carried out on the QIAsymphony SP robot using QIAsymphony DSP DNA Midi Kit (QIAGEN GmbH, Hilden, Germany). All samples were checked for purity and normalized to a standard concentration of 100 ng/μl. Whole‐exome sequencing was performed by BGI Shenzhen, Guangdong, China. BGI Exome (59 M) capture kit was used for library capture and sequencing performed using the Illumina HiSeq platform (Illumina, San Diego, USA). Sequence reads for each sample were aligned to the reference genome (GRCh37 (hg19)) using Burrows‐Wheeler Aligner BWA V0.7.15 (Li & Durbin, 2009). Variant calls were hard filtered according to quality control metrics: Quality by depth, Fisher strand (to detect strand bias), RMS Mapping quality, and Read position rank sum (testing for distance from the end of read). Annotation included the assignment of amino acid changes, gnomAD allele frequency, and functional prediction score from CADD (Rentzsch, Witten, Cooper, Shendure, & Kircher, 2019). Variant filtering was carried out to highlight rare (minor allele frequency <0.001) and damaging variants segregating with disease status within the pedigree. Filtered variants were classified according to the American College of Medical Genetics and Genomics (ACMG) guidelines (Richards et al., 2015). The *NOTCH1* (NM_017617.5) variant was confirmed by PCR and Sanger sequencing. Figure 1b Sanger sequencing was performed by Source Bioscience, Nottingham, UK; PCR Forward Primer: 5′‐CCTTCAGCACCCCACTCAG‐3′, PCR Reverse primer: 5′‐CCGTGACACTTGGGACGTTC‐3′ (Eurofins, Ebersberg, Germany).

## 3 RESULTS

The genetic family tree is presented in Figure 1a.The Proband was a 24‐year‐old Caucasian male attending a regular follow up in the Adult Congenital Heart Clinic of the East Midlands Congenital Heart Centre, Leicester, UK. He had undergone an open heart repair of a large sub‐aortic VSD at the age of one year. Cardiac MRI performed at the age of 28 years showed good long term results of VSD repair, well‐functioning BAV with right and left cusp fusion pattern, and dilatation of the aortic root (39 mm) and ascending aorta (40 mm) (Figure 1c and d). The proband's mother underwent aortic valve replacement for severe aortic valve stenosis at the age of 50 years. She had undergone a CT angiogram of her aorta at the age of 46 years, which showed heavily calcified, probably tri‐leaflet aortic valve, dilatation of the aortic root (38 mm), and ascending aorta (44 mm) (Figure 1e and f). Pre‐operational trans‐oesophageal echocardiogram identified the valve as tri‐leaflet with severe calcification, severe stenosis, and moderate incompetence. The maternal grandmother underwent aortic valve replacement with a mechanical aortic valve prosthesis and simultaneous coronary artery bypass surgery at the age of 55 due to severe aortic valve stenosis and obstructive coronary artery disease. The most recent transthoracic echocardiogram performed at the age of 72 years showed no evidence of aortic root or ascending aorta dilatation. The proband's maternal grandfather underwent transthoracic echocardiogram as a part of the study recruitment protocol. This showed normally functioning tri‐leaflet aortic valve.

Exome sequencing, which was performed in the four family members with known affection status (proband, affected mother, affected maternal grandmother, and unaffected maternal grandfather), identified 16 rare, potentially damaging, genetic variants comprising two potential loss of function variants (one stop and one splice variant), and 14 missense variants, co‐segregating with the aortic valve disease phenotype (Table 1). We classified each variant in accordance with the ACMG guidelines and identified A c.873C>G/p.Tyr291* stop mutation in *NOTCH1*, the most deleterious variant in our analysis, as causative.

**Table 1:** Rare, potentially damaging, genetic variants co‐segregating with affection status in the presented pedigree

Variant ID	Gene	OMIM ID	MAF (gnomAD)	Function	CADD score (PHRED)	ACMG classification
c.873C>G/p.Tyr291*	*NOTCH1*	190198	NA	Stop gained	39	Pathogenic
rs200827136	*ADAMTS16*	607510	0.0004	Missense	32	Uncertain significance
rs193181260	*PCNX2*	617656	0.0001	Missense	32	Likely benign
rs149425237	*PLOD1*	153454	0.0005	Missense	28.5	Likely benign
c.2849A>G/p.Glu950Gly	*CNTNAP2*	604569	NA	Missense	28.6	Uncertain significance
rs763341735	*CDH15*	114019	0.00002	Missense	25.1	Benign
c.2237C>T/p.Ser746Phe	*ANKRD27*	NA	NA	Missense	26.3	Uncertain significance
rs754855234	*CTTNBP2*	609772	0.00004	Missense	24.7	Uncertain significance
c.2236T>G /p.Tyr746Asp	*CNTN2*	190197	NA	Missense	26.4	Uncertain significance
rs201232704	*PHRF1*	611780	0.00009	Missense	23.6	Likely benign
rs765953991	*KDM6A*	300128	0.00001	Missense	27.1	Uncertain significance
rs371508867	*ADGRL3*	616417	0.00002	Missense	24.9	Uncertain significance
rs377579773	*CADPS2*	609978	0.00004	Missense	26.6	Uncertain significance
rs370275584	*NOC3L*	610769	0.0001	Missense	24.1	Uncertain significance
rs370153408	*PRKDC*	600899	0.00003	Missense	23.8	Likely benign
c.930+1G>C	*ANKRD36*	NA	NA	Splice donor variant +intron variant	20.8	Likely benign

Note

CADD, combined annotation dependent depletion score; Gene, gene symbol; ID, identifier; MAF, genome aggregation database (gnomAD) minor allele frequency; NA, not available.

*NOTCH1* loss of function mutations has previously been reported to cause BAV (Garg et al., 2005; Kerstjens‐Frederikse et al., 2016) and Tyr291*, which is encoded by *NOTCH1* exon 6 and located in the 7^th^ EGFR‐like extracellular domain of NOTCH1 (Figure S1).

## 4 DISCUSSION

Herein we present a nuclear family affected by a complex cardiac phenotype comprising a combination of BAV, aortic valve stenosis, TAA, and VSD. The proband presents the most common type of BAV with the fusion of the right and left valve leaflets and visible raphe. Consistent with this type of BAV leaflet fusion is the dilatation of the aortic root observed in the patient (Miskowiec et al., 2018). Both in the maternal grandmother and the proband's mother, the stenosed aortic valves were described as trileaflet and heavily calcified. Ascending aorta dilatation is present in the proband and his mother but not in the maternal grandmother. It can only be speculated whether the lack of dilatation of the thoracic aorta in the grandmother might be a result of favorable hemodynamic function of the aortic valve, incomplete penetrance of the *NOTCH1* mutation or other genetic/environmental modifiers (Hope et al., 2012). The co‐existence of BAV and VSD (observed in the proband) has been previously reported in the literature; however, no accurate estimate of the prevalence of the combined lesion exists (Duran et al., 1995). The clinical picture observed in the presented pedigree is consistent with previous reports on clinical phenotypes associated with inactivating *NOTCH1* mutations (Kerstjens‐Frederikse et al., 2016; Southgate et al., 2015). The majority of congenital lesions present in patients with inactivating *NOTCH1* mutations group around the left ventricular outflow as in the reported pedigree. Mutations in *NOTCH1* have been reported to associate with both familial and sporadic forms of BAV. However, to our knowledge, inactivating *NOTCH1* mutations leading to *NOTCH1* haploinsufficiency have almost exclusively been reported in the familial form of BAV disease (Gillis et al., 2017; Kerstjens‐Frederikse et al., 2016).

To our knowledge, the p.Tyr291* *NOTCH1* mutation, cosegregating with the phenotype within our pedigree, is the most proximal stop mutation reported to date (Figure S1) It is possible that this early stop codon leads to nonsense‐mediated decay. This, in turn, would effectively lead to *NOTCH1* haploinsufficiency in the carriers of the mutation. Alternatively, the *NOTCH1* allele containing the premature stop produces a truncated 291 amino‐acid long polypeptide chain. The stop codon is localized within the seventh EGFR‐like extracellular domain. NOTCH1 contains 36 EGFR‐like domains in the extracellular portion of the receptor with the ligand‐binding site formed by domains 11–13 (Hambleton et al., 2004). The receptor lacking EGFR‐like domains 11–13 would, therefore, not be able to interact with ligands or induce signaling and would, therefore, be functionally inactive (Ge, Liu, Hou, & Stanley, 2008).

The exact pathogenesis of BAV remains elusive. NOTCH pathways are important regulators of cell fate decisions, proliferation, and apoptosis during embryonic development and in adult life (Meester et al., 2019). The NOTCH1 pathway is directly involved in the development of the valve‐forming fields during cardiogenesis and is crucial for the correct development of endocardial cushions through regulation of endocardial to mesenchymal transformation and remodeling of the immature aortic valve (Luna‐Zurita et al., 2010, Koenig et al., 2016; Timmerman et al., 2004).

Accumulating evidence links NOTCH1 signaling with the development of aortic valve stenosis. NOTCH1 plays an important role in response to valve hemodynamic injury and stress (Chen et al., 2015). Several studies have suggested a protective role for NOTCH1 signaling in the prevention of aortic valve calcification by repressing activation of pro‐osteogenic signaling within the aortic valve (Acharya et al., 2011; Chen et al., 2015; Nigam & Srivastava, 2009) TAAs have also been consistently observed in pedigrees with inactivating *NOTCH1* mutations (Kerstjens‐Frederikse et al., 2016). TAA is a common phenotype associated with BAV and requires surgical intervention in one in four patients with BAV over 25 years of observation (Michelena et al., 2011). Accumulating evidence suggest that NOTCH1 apart from its role in vasculogenesis also plays a significant role in the pathological remodelling of aorta and development TAA (Malashicheva et al., 2020). NOTCH1 pathway has been shown to be downregulated in the aortic tissue of patients with BAV (Balistreri et al., 2018). In addition, *Notch1* haploinsufficiency in mice can lead to the development of ascending aortic dilatation accompanied by histological changes in the aortic media typical of TAA (Koenig et al., 2017).

In view of the above evidence, the presence of a damaging *NOTCH1* mutation in the presented pedigree provides the likely biological explanation for the occurrence of BAV, sub‐aortic VSD, aortic valve calcification, and TAA in the presented pedigree. The identification and reporting of the genetic causes of BAV will lead to a better understanding of both disease pathogenesis and the development of comorbidities such as aortic stenosis and may lead to the development of effective therapies.

## CONFLICT OF INTEREST

The authors declare no conflict of interest.

## AUTHOR CONTRIBUTIONS

Radoslaw Debiec and Stephen E. Hamby—Study concept and design, analysis of data, drafting of the manuscript. Peter Jones and Gregory J. Skinner—acquisition of data, analysis and interpretation of data, critical revision. Sue Coolman, Manish Asiani and Shireen Kharodia—acquisition of data, critical revision. Nilesh J. Samani, Tom R. Webb and Aidan Bolger—study concept and design, analysis and interpretation of data, critical revision.

## Supporting information

Fig S1

## References

[mgg31437-bib-0001] Acharya, A. , Hans, C. P. , Koenig, S. N. , Nichols, H. A. , Galindo, C. L. , Garner, H. R. , … Garg, V. (2011). Inhibitory role of Notch1 in calcific aortic valve disease. PLoS One, 6(11), e27743 10.1371/journal.pone.0027743 22110751 PMC3218038

[mgg31437-bib-0002] Balistreri, C. R. , Crapanzano, F. , Schirone, L. , Allegra, A. , Pisano, C. , Ruvolo, G. , … Frati, G. (2018). Deregulation of Notch1 pathway and circulating endothelial progenitor cell (EPC) number in patients with bicuspid aortic valve with and without ascending aorta aneurysm. Scientific Reports, 8(1), 13834 10.1038/s41598-018-32170-2 30218064 PMC6138685

[mgg31437-bib-0003] Chen, J. , Ryzhova, L. M. , Sewell‐Loftin, M. K. , Brown, C. B. , Huppert, S. S. , Baldwin, H. S. , & Merryman, W. D. (2015). Notch1 mutation leads to valvular calcification through enhanced myofibroblast mechanotransduction. Arteriosclerosis, Thrombosis, and Vascular Biology, 35(7), 1597–1605. 10.1161/ATVBAHA.114.305095 PMC4600070 26023079

[mgg31437-bib-0004] Ducharme, V. , Guauque‐Olarte, S. , Gaudreault, N. , Pibarot, P. , Mathieu, P. , & Bosse, Y. (2013). NOTCH1 genetic variants in patients with tricuspid calcific aortic valve stenosis. Journal of Heart Valve Disease, 22(2), 142–149. Retrieved from http://www.ncbi.nlm.nih.gov/pubmed/23798201 23798201

[mgg31437-bib-0005] Duran, A. C. , Frescura, C. , Sans‐Coma, V. , Angelini, A. , Basso, C. , & Thiene, G. (1995). Bicuspid aortic valves in hearts with other congenital heart disease. Journal of Heart Valve Disease, 4(6), 581–590. Retrieved from http://www.ncbi.nlm.nih.gov/pubmed/8611973 8611973

[mgg31437-bib-0006] Freylikhman, O. , Tatarinova, T. , Smolina, N. , Zhuk, S. , Klyushina, A. , Kiselev, A. , … Kostareva, A. (2014). Variants in the NOTCH1 gene in patients with aortic coarctation. Congenital Heart Disease, 9(5), 391–396. 10.1111/chd.12157 24418111

[mgg31437-bib-0007] Garg, V. , Muth, A. N. , Ransom, J. F. , Schluterman, M. K. , Barnes, R. , King, I. N. , … Srivastava, D. (2005). Mutations in NOTCH1 cause aortic valve disease. Nature, 437(7056), 270–274. 10.1038/nature03940 16025100

[mgg31437-bib-0008] Ge, C. , Liu, T. , Hou, X. , & Stanley, P. (2008). In vivo consequences of deleting EGF repeats 8–12 including the ligand binding domain of mouse Notch1. BMC Developmental Biology, 8, 48 10.1186/1471-213X-8-48 18445292 PMC2390518

[mgg31437-bib-0009] Gillis, E. , Kumar, A. A. , Luyckx, I. , Preuss, C. , Cannaerts, E. , van de Beek, G. , … Loeys, B. L. (2017). Candidate gene resequencing in a large bicuspid aortic valve‐associated thoracic aortic aneurysm cohort: SMAD6 as an important contributor. Frontiers in Physiology, 8, 400 10.3389/fphys.2017.00400 28659821 PMC5469151

[mgg31437-bib-0010] Gould, R. A. , Aziz, H. , Woods, C. E. , Seman‐Senderos, M. A. , Sparks, E. , Preuss, C. , … Dietz, H. C. (2019). ROBO4 variants predispose individuals to bicuspid aortic valve and thoracic aortic aneurysm. Nature Genetics, 51(1), 42–50. 10.1038/s41588-018-0265-y 30455415 PMC6309588

[mgg31437-bib-0011] Hambleton, S. , Valeyev, N. V. , Muranyi, A. , Knott, V. , Werner, J. M. , McMichael, A. J. , … Downing, A. K. (2004). Structural and functional properties of the human notch‐1 ligand binding region. Structure, 12(12), 2173–2183. 10.1016/j.str.2004.09.012 15576031

[mgg31437-bib-0012] Helle, E. , Cordova‐Palomera, A. , Ojala, T. , Saha, P. , Potiny, P. , Gustafsson, S. , … Priest, J. R. (2019). Loss of function, missense, and intronic variants in NOTCH1 confer different risks for left ventricular outflow tract obstructive heart defects in two European cohorts. Genetic Epidemiology, 43(2), 215–226. 10.1002/gepi.22176 30511478 PMC6375786

[mgg31437-bib-0013] Hope, M. D. , Wrenn, J. , Sigovan, M. , Foster, E. , Tseng, E. E. , & Saloner, D. (2012). Imaging biomarkers of aortic disease: increased growth rates with eccentric systolic flow. Journal of the American College of Cardiology, 60(4), 356–357. 10.1016/j.jacc.2012.01.072 22813616

[mgg31437-bib-0014] Kerstjens‐Frederikse, W. S. , van de Laar, I. M. B. H. , Vos, Y. J. , Verhagen, J. M. A. , Berger, R. M. F. , Lichtenbelt, K. D. , … Wessels, M. W. (2016). Cardiovascular malformations caused by NOTCH1 mutations do not keep left: data on 428 probands with left‐sided CHD and their families. Genetics in Medicine, 18(9), 914–923. 10.1038/gim.2015.193 26820064

[mgg31437-bib-0015] Koenig, S. N. , Bosse, K. , Majumdar, U. , Bonachea, E. M. , Radtke, F. , & Garg, V. (2016). Endothelial Notch1 is required for proper development of the semilunar valves and cardiac outflow tract. Journal of the American Heart Association, 5(4), e003075 10.1161/JAHA.115.003075 27107132 PMC4843530

[mgg31437-bib-0016] Koenig, S. N. , LaHaye, S. , Feller, J. D. , Rowland, P. , Hor, K. N. , Trask, A. J. , … Garg, V. (2017). Notch1 haploinsufficiency causes ascending aortic aneurysms in mice. JCI Insight, 2(21), e91353 10.1172/jci.insight.91353 PMC5752295 29093270

[mgg31437-bib-0017] Li, H. , & Durbin, R. (2009). Fast and accurate short read alignment with Burrows‐Wheeler transform. Bioinformatics, 25(14), 1754–1760. 10.1093/bioinformatics/btp324 19451168 PMC2705234

[mgg31437-bib-0018] Luna‐Zurita, L. , Prados, B. , Grego‐Bessa, J. , Luxán, G. , del Monte, G. , Benguría, A. , … de la Pompa, J. L. (2010). Integration of a Notch‐dependent mesenchymal gene program and Bmp2‐driven cell invasiveness regulates murine cardiac valve formation. Journal of Clinical Investigation, 120(10), 3493–3507. 10.1172/JCI42666 20890042 PMC2947227

[mgg31437-bib-0019] Malashicheva, A. , Kostina, A. , Kostareva, A. , Irtyuga, O. , Gordeev, M. , & Uspensky, V. (2020). Notch signaling in the pathogenesis of thoracic aortic aneurysms: A bridge between embryonic and adult states. Biochimica et Biophysica Acta (BBA)—Molecular Basis of Disease, 1866(3), 165631 10.1016/j.bbadis.2019.165631 31816439

[mgg31437-bib-0020] Meester, J. A. N. , Verstraeten, A. , Alaerts, M. , Schepers, D. , Van Laer, L. , & Loeys, B. L. (2019). Overlapping but distinct roles for NOTCH receptors in human cardiovascular disease. Clinical Genetics, 95(1), 85–94. 10.1111/cge.13382 29767458

[mgg31437-bib-0021] Michelena, H. I. , Desjardins, V. A. , Avierinos, J.‐F. , Russo, A. , Nkomo, V. T. , Sundt, T. M. , … Enriquez‐Sarano, M. (2008). Natural history of asymptomatic patients with normally functioning or minimally dysfunctional bicuspid aortic valve in the community. Circulation, 117(21), 2776–2784. 10.1161/CIRCULATIONAHA.107.740878 18506017 PMC2878133

[mgg31437-bib-0022] Michelena, H. I. , Khanna, A. D. , Mahoney, D. , Margaryan, E. , Topilsky, Y. , Suri, R. M. , … Enriquez‐Sarano, M. (2011). Incidence of aortic complications in patients with bicuspid aortic valves. JAMA, 306(10), 1104–1112. 10.1001/jama.2011.1286 21917581

[mgg31437-bib-0023] Miśkowiec, D. , Lipiec, P. , Szymczyk, E. , Wejner‐Mik, P. , Michalski, B. , Kupczyńska, K. , … Kasprzak, J. D. (2018). Bicuspid aortic valve morphology and its impact on aortic diameters‐A systematic review with meta‐analysis and meta‐regression. Echocardiography, 35(5), 667–677. 10.1111/echo.13818 29399873

[mgg31437-bib-0024] Mordi, I. , & Tzemos, N. (2012). Bicuspid aortic valve disease: A comprehensive review. Cardiology Research and Practice, 2012, 196037 10.1155/2012/196037 22685681 PMC3368178

[mgg31437-bib-0025] Nigam, V. , & Srivastava, D. (2009). Notch1 represses osteogenic pathways in aortic valve cells. Journal of Molecular and Cellular Cardiology, 47(6), 828–834. 10.1016/j.yjmcc.2009.08.008 19695258 PMC2783189

[mgg31437-bib-0026] Rentzsch, P. , Witten, D. , Cooper, G. M. , Shendure, J. , & Kircher, M. (2019). CADD: Predicting the deleteriousness of variants throughout the human genome. Nucleic Acids Research, 47(D1), D886–D894. 10.1093/nar/gky1016 30371827 PMC6323892

[mgg31437-bib-0027] Richards, S. , Aziz, N. , Bale, S. , Bick, D. , Das, S. , Gastier‐Foster, J. , … Rehm, H. L. (2015). Standards and guidelines for the interpretation of sequence variants: A joint consensus recommendation of the American College of Medical Genetics and Genomics and the Association for Molecular Pathology. Genetics in Medicine, 17(5), 405–424. 10.1038/gim.2015.30 25741868 PMC4544753

[mgg31437-bib-0028] Southgate, L. , Sukalo, M. , Karountzos, A. S. V. , Taylor, E. J. , Collinson, C. S. , Ruddy, D. , … Trembath, R. C. (2015). Haploinsufficiency of the NOTCH1 receptor as a cause of adams‐oliver syndrome with variable cardiac anomalies. Circulation‐Cardiovascular Genetics, 8(4), 572–581. 10.1161/Circgenetics.115.001086 25963545 PMC4545518

[mgg31437-bib-0029] Timmerman, L. A. , Grego‐Bessa, J. , Raya, A. , Bertran, E. , Perez‐Pomares, J. M. , Diez, J. , … de la Pompa, J. L. (2004). Notch promotes epithelial‐mesenchymal transition during cardiac development and oncogenic transformation. Genes & Development, 18(1), 99–115. 10.1101/gad.276304 14701881 PMC314285

