# Supplementary material for: Novel loss of function mutation in NOTCH1 in a family with bicuspid aortic valve, ventricular septal defect, thoracic aortic aneurysm, and aortic valve stenosis
Source: Mol Genet Genomic Med. 2020 Jul 27;8(10):e1437. doi: 10.1002/mgg3.1437 (PMC7549557; doi:10.1002/mgg3.1437)
Supplement: Supplementary file 1 — Fig S1 [file MGG3-8-e1437-s001.pdf]

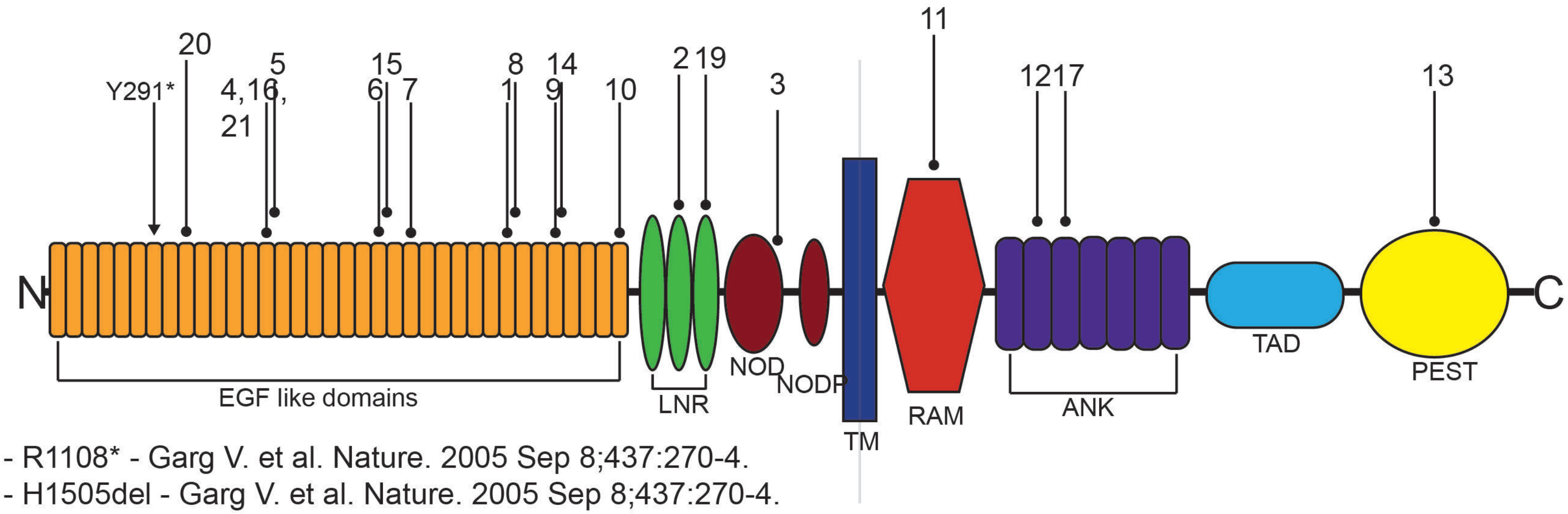

1 - R1108* - Garg V. et al. Nature. 2005 Sep 8;437:270-4.
2 - H1505del - Garg V. et al. Nature. 2005 Sep 8;437:270-4.
3 - Y1619* - Foffa I. et al. BMC Med Genet. 2013;14:44.
4 - Y550* - Kerstjens-Frederikse WS et al. Genet Med. 2016;18:914-23.
5 - G557fs - Kerstjens-Frederikse WS et al. Genet Med. 2016;18:914-23.
6 - D809Tfs*67 - Kerstjens-Frederikse WS et al. Genet Med. 2016;18:914-23.
7 - A882Hfs*297 - Kerstjens-Frederikse WS et al. Genet Med. 2016;18:914-23.
8 - C1018* - Kerstjens-Frederikse WS et al. Genet Med. 2016;18:914-23.
9 - E1262_G1301del - Kerstjens-Frederikse WS et al. Genet Med. 2016;18:914-23.
10 - C1414Afs*31 - Kerstjens-Frederikse WS et al. Genet Med. 2016;18:914-23.
11 - W1843* - Kerstjens-Frederikse WS et al. Genet Med. 2016;18:914-23.
12 - R1984* - Kerstjens-Frederikse WS et al. Genet Med. 2016;18:914-23.
13 - S2486Lfs*21 - Kerstjens-Frederikse WS et al. Genet Med. 2016;18:914-23.
14 - C1255* - Preuss C et al. PLoS Genet. 2016 Oct 19;12(10):e1006335.
15 - Y813*- Preuss C et al. PLoS Genet. 2016 Oct 19;12(10):e1006335.
16 - Y550* - Southgate L et al. Circ Cardiovasc Genet. 2015;8:572-581.
17 - S2017Tfs*9 - Southgate L et al. Circ Cardiovasc Genet. 2015;8:572-581.
18 - c.743-1F>T - Stitrich AB et al. Am J Hum Genet. 2014 Sep 4;95(3):275-84.
19 - C1554* Durbin MD et al. Pediatr Cardiol. 2017;38(6):1232-1240.
20 - C359* Helle E. et al. Genet Epidemiol. 2019;43:215-226.
21 - Y550* Helle E. et al. Genet Epidemiol. 2019;43:215-226.
22 - R1761Gfs*37 Jia Y et al. Am J Med Genet A 167A:1822–1829.
